# The Use of Effective Core Potentials with Multiconfiguration Pair-Density Functional Theory

**DOI:** 10.1021/acs.jpca.4c02666

**Published:** 2024-07-25

**Authors:** William E. Minnette, Erik P. Hoy, Andrew M. Sand

**Affiliations:** † Department of Chemistry and Biochemistry, Butler University, Indianapolis, Indiana 46208, United States; ‡ Department of Chemistry, Rowan University, Glassboro, New Jersey 08028, United States

## Abstract

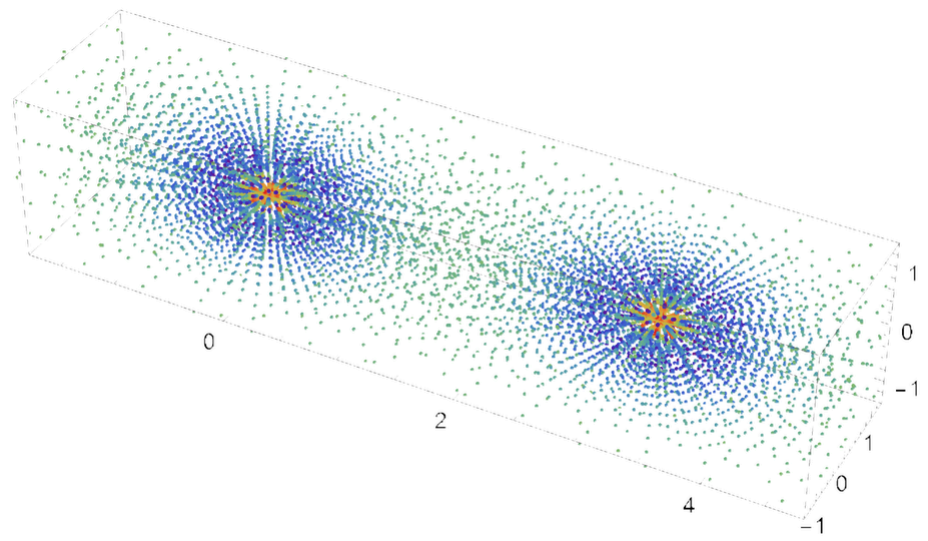

The reliable and accurate prediction of chemical properties is a key goal in quantum chemistry. Transition-metal-containing complexes can often pose difficulties to quantum mechanical methods for multiple reasons, including many electron configurations contributing to the overall electronic description of the system and the large number of electrons significantly increasing the amount of computational resources required. Often, multiconfigurational electronic structure methods are employed for such systems, and the cost of these calculations can be reduced by the use of an effective core potential (ECP). In this work, we explore both theoretical considerations and performances of ECPs applied in the context of multiconfiguration pair-density functional theory (MC-PDFT). A mixed-basis set approach is used, using ECP basis sets for transition metals and all-electron basis sets for nonmetal atoms. We illustrate the effects that an ECP has on the key parameters used in the computation of MC-PDFT energies, and we explore how ECPs affect the prediction of physical observables for chemical systems. The dissociation curve for a metal dimer was explored, and ionization energies for transition metal-containing diatomic systems were computed and compared to experimental values. In general, we find that ECP approaches employed with MC-PDFT are able to predict ionization energies with improved accuracy compared to traditional Kohn–Sham density functional theory approaches.

## 1 Introduction

An accurate description of the electronic structure for transition metal-containing compounds is important to study due to the critical role these compounds play in a number of important chemical systems,^1^ including biological systems,^2^ catalysts in synthetic chemistry,^3,4^ and molecular electronic devices.^5−7^ The degeneracy or near degeneracy of the d orbitals often leads to significant multireference (strong) correlation energy, and the theoretical study of strongly correlated systems is challenging^8^ especially as the size of the system (number of electrons) increases which can quickly make some calculations intractable. Treating such systems requires specialized methodologies including (but not limited to) multireference perturbation theories,^9−12^ multireference configuration interaction approaches,^13^ and multireference density functional theory approaches.^14,15^

Multiconfiguration pair-density functional theory (MC-PDFT)^14^ is a density-functional-based approach to describing the electronic structure and chemical properties of highly correlated chemical systems. In an MC-PDFT calculation, an on-top density functional is used to calculate an improved electronic energy from a reference calculation, most typically a complete active space self-consistent field (CASSCF) calculation. The on-top density functional is a functional of the one-electron density, the two-electron on-top pair density (related to the probability of finding two electrons on top of each other in the same region of space), and their derivatives. MC-PDFT has demonstrated the ability to predict many important chemical properties with an accuracy comparable to many wave function-based post-CASSCF approaches (including CASPT2) at a significantly lower computational cost.^16^

Despite the lower cost of MC-PDFT compared to other CASSCF methods, treating transition metal containing systems is still computationally costly, particularly for systems with a large number of metal atoms such as molecules attached to an electrode. Effective core potentials (ECPs) provide a well-tested and reliable avenue for the acceleration of computational calculations containing heavy atoms. With an ECP, the explicit treatment of selected core electrons is replaced by a potential energy function, thereby reducing the number of basis functions needed in the electronic structure calculation. Additional effects, such as relativistic effects, can be included in the definition of the ECP, thereby avoiding the associated computational costs with a relativistic all-electron electronic structure calculation. The treatment of chemically inactive core electrons with an ECP reduces the computational cost of a calculation, as these calculations will scale polynomially with the number of explicitly correlated (non-ECP) electrons.

Some of the most popularly employed ECPs include the Los Alamos National Laboratory 2 double-ζ (LANL2DZ)^17−19^ and the Stuttgart-Dresden-Bonn energy consistent pseudopotentials (often denoted MDF10 in the literature).^20,21^ Many popular ECPs have been developed in the context of Hartree–Fock (HF) calculations, whereby ECP parameters are optimized to best reproduce experimental excitation and ionization energies.^20^ In practice, ECPs are used in a wide variety of wave function and density functional theory approaches beyond the HF level, and the transferability of these ECPs to correlated methods has been explored for wave function-based and density functional theory (DFT)-based calculations.^22−25^ Additionally, some references have developed ECPs for specific use in DFT.^26−28^ In the case of DFT, issues can arise due to the nonlinear dependence of the exchange-correlation functional on the electron density.^29,30^ A number of recent studies have explored the performance of pseudopotential approaches in density functional theory.^25,27,28,31,32^ Results of previous works highlight that “off-the-shelf” ECP prescriptions can often result in significant errors in the calculation of many molecular properties, including bond energies and excitation energies. Additionally, other groups have also explored the performance of mixed basis set approaches, with the assembly of a compact effective potential with valence basis sets.^33,34^ Together, these studies demonstrate the importance of thoroughly investigating potential ECP errors when pairing them with a methodology for the first time.

Previous works have employed ECPs in the context of MC-PDFT, including a calculation of the silver dimer potential energy curve.^35^ To our knowledge, however, no previous study has explored the mathematical effects that the application of the ECP has on the computation of the on-top energy functional. A previous study has explored how the on-top ratio changes as a function of active space selections, which in turn affects the calculated on-top energy portion of the MC-PDFT energy.^36^ In particular, this work showed that the on-top energy was slow to converge with an increase in active space size. In the most widely used approach, MC-PDFT calculations are based on the translation of GGA functionals, and both the electron density and density gradient are ingredients in the density functional. The description of the core electrons by an ECP will alter both of these values, and this could impact the performance of MC-PDFT. Further, no previous works have evaluated the performance of MC-PDFT with commonly applied ECP bases in the computation of chemical properties. The use of ECP bases in the context of MC-PDFT is important, with MC-PDFT-based theories being applied to large systems in which ECP basis sets can facilitate with computational cost.^37^

In this work, we evaluate the performance of using commonly employed ECPs in conjunction with MC-PDFT. First, we address the mechanistic and theoretical effects that ECP basis sets have on the computation of the MC-PDFT energy and the on-top pair density. Then, we quantify how the addition of ECPs to an MC-PDFT approach impacts the ability of the method to predict certain experimental observables, including accurate potential energy surfaces, equilibrium bond distances, and ionization energies for first-row transition metal species. Limiting our initial analysis to first-row transition metals allows for a more direct evaluation of the effect of the ECP on the result as non-ECP basis sets are readily available for these particular atoms.

## 2 Theory

### 2.1 MC-PDFT Theory

An MC-PDFT energy is computed via the following equation:^14,38^
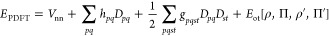
1where *h*_*pq*_ and *g*_*pqst*_ represent the one- and two-electron integrals:
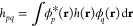
2
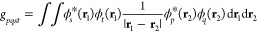
3where the one-electron operator *h*(**r**) accounts for both electronic kinetic energy and electron–nuclear potential energy:
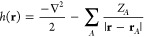
4Molecular orbitals are indicated by ϕ_*i*_; ρ and Π are the electronic density and the on-top pair density, respectively, and ρ′ and Π′ are their derivatives. These densities can be expressed in terms of the orbitals, the one-body density matrix *D*, and the two-body density matrix *d*:
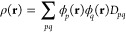
5
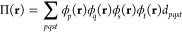
6

The most commonly employed functionals in MC-PDFT are translated (denoted by a “t”), in which *E*_ot_[ρ, Π, ρ′, Π′] is formed by translating existing local density approximation and generalized gradient approximation Kohn–Sham (KS) density functionals.^14,39^ The parent KS density functionals depend on the spin-up and spin-down electron densities ρ_α_ and ρ_β_ as well as their spatial derivatives ρ_α_′ and ρ_β_′. We express these KS functionals as *E*_xc_[ρ_α_, ρ_β_, ρ_α_′, and ρ_β_′]. This translation scheme^14^ defines the on-top energy functional (with no dependence on Π′) as
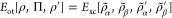
7where the translated densities are defined as
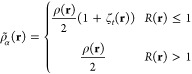
8
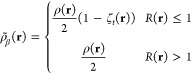
9
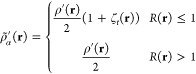
10
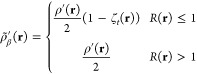
11where
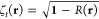
12
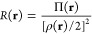
13and
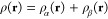
14
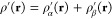
15*R*(**r**) is the on-top ratio. This term serves to quantify the difference between an uncorrelated single-determinant representation and the actual correlated density and the top density used from the reference wave function. For a single-determinant case, the value of the on-top ratio is 1 at all points. For correlated representations, this value can deviate from 1, either larger or smaller. As evidenced by eqs 8–11, points where this ratio is less than 1 results in a translation of the densities in the KS functional. When an ECP is employed in lieu of an all-electron basis set, the two important elements (density and pair density) that determine the on-top ratio will change as the number of explicit electrons are reduced. A key question explored in this work is how these changes, in turn, affect the on-top ratio and the ability of the MC-PDFT functional to recover correlation energy.

## 3 Methods

This work explores two concepts: (i) the mechanistic/theoretical effects of employing an ECP with MC-PDFT and (ii) the performance of MC-PDFT in the determination of bond lengths, geometries, and excitation energies using ECPs. All calculations were performed using the OpenMolcas software package.^40^

To understand the mechanistic and theoretical effects, a series of calculations was performed on the chromium dimer system. All MC-PDFT calculations employed a (12e, 12o) active space CASSCF calculation as a reference wave function. Near-equilibrium (1.75 Å) and stretched (2.10 Å) geometries were explored. The dev2-SVP all-electron basis set^41^ and Stuttgart RSC 1997 ECP basis set^20,21^ (referred to as “Stuttgart” throughout this work) were used, as well as a combined case where the ECP-only part of the Stuttgart basis set was included with the dev2-SVP basis set. Plots of the on-top ratio for each basis set were generated by taking a one-dimensional cross section along the bonding axis.

To illustrate how energy is affected in different regions in space by the inclusion of an ECP, three-dimensional plots were produced by comparing two MC-PDFT calculations using an identical grid and all-electron (cc-pVDZ^42−44^) and ECP-containing (Stuttgart RSC 1997) basis sets. The weighted energy at each gridpoint was individually normalized for each basis set, and the difference between the two sets of gridpoints was computed and plotted. This results in an image that identifies the regions of space where the relative energy contribution to the total MC-PDFT energy changes with the use of a full-electron or an ECP basis set.

To explore complete bond dissociation, MC-PDFT was applied to a copper dimer system with ECP-containing basis sets and all-electron basis sets. These calculations employed a (2e, 2o) active space CASSCF calculation, and different combinations of functionals (tPBE, ftPBE), relativistic versus nonrelativistic Hamiltonians (and associated basis sets), and ECP-based basis sets (Stuttgart and LANL2DZ^17−19^) were used.

Finally, the performance of MC-PDFT with the use of ECP basis sets was explored in the determination of adiabatic excitation energies for a set of transition-metal-containing dimers. A commonly used mixed-basis set approach was used, employing ECP basis sets for the transition metal atoms and the maug-cc-pVDZ basis set^45^ for the nontransition metal atoms. CASSCF calculations were used as the reference wave functions, and details of the active spaces are given in the Results and Discussion section.

## 4 Results and Discussion

### 4.1 ECPs and the On-Top Pair Energy

In order to understand the effect of how an effective core potential affects the ability of the MC-PDFT approach to recover correlation energy, calculations were performed on the chromium dimer system with an active space of (12e,12o). Three different basis sets were explored: the traditional dev2-SVP basis set without an ECP, the Stuttgart basis with an ECP (unmodified), and the dev2-SVP basis set, in which the 10-electron effective core potential from the Stuttgart ECP basis set was added. Plots of the on-top ratio, the one-electron density, and the two-electron density along the bonding axis are shown for an atomic separation of 1.75 Å and 2.10 Å. In each plot, one chromium atom is located at the origin (position 0 on the bonding axis).

**Figure 1:**
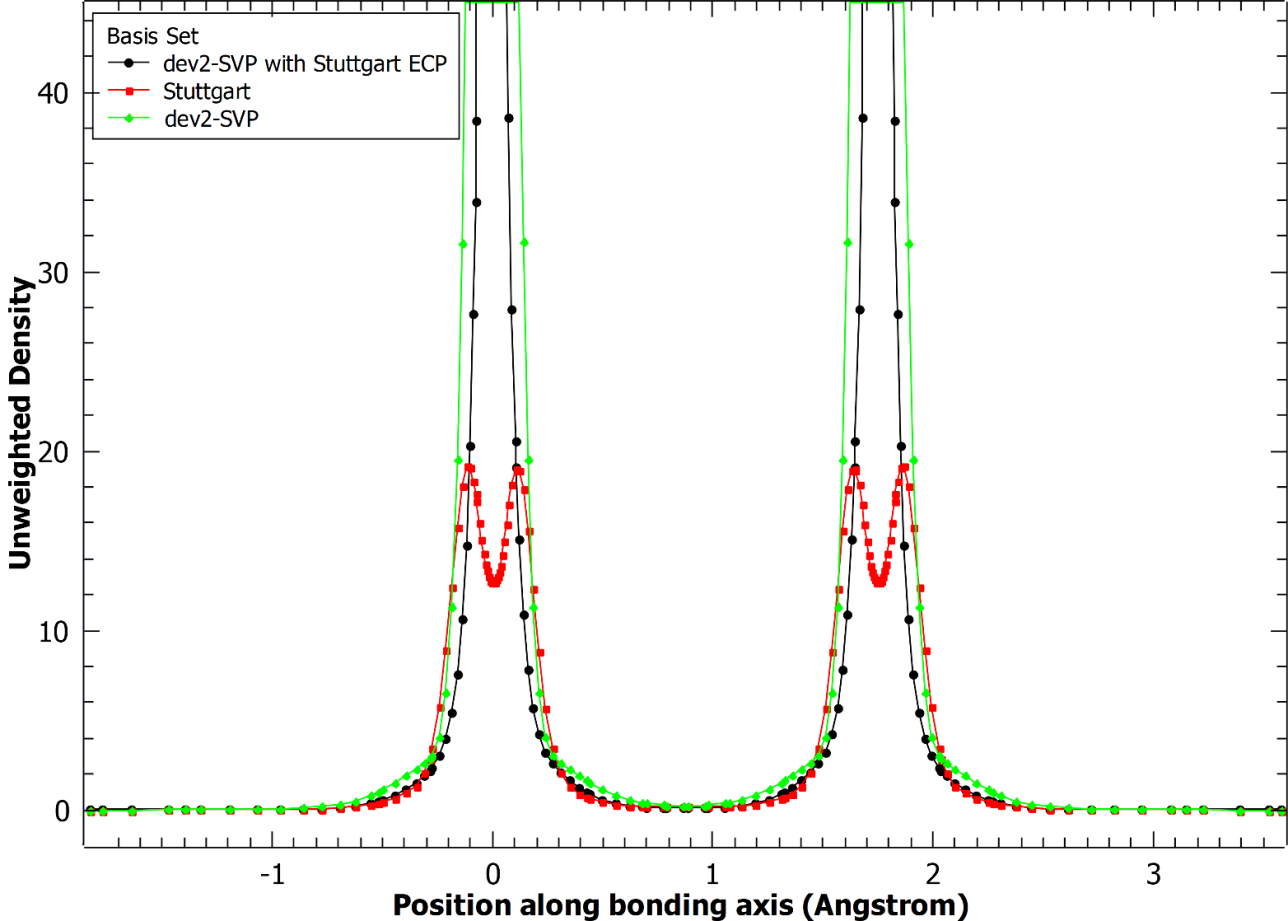
Unweighted electron density along the bonding axis for the chromium dimer with and without an effective core potential applied. Cr atoms are 1.75 Å apart.

We begin by examining the effect of the ECP basis set on the underlying CASSCF reference calculation. Two components from the reference calculation are needed in the determination of the MC-PDFT energy: the density and pair density. First, we explored the reference density (Figure 1) with an atomic separation of 1.75 Å, which is very close to the experimental bond distance. An expansive grid is used, with some points more than 30 Å away from the nuclear centers, but these plots only show the grid points near the nuclei that contribute most significantly to the total electronic energy. In the density, we see that the unmodified Stuttgart ECP reduces the density near the nucleus, as expected. Some small differences in the density are seen in some intermediate distances (0.2–0.5 Å, for example). These differences might be attributed to the ECP, but they may be due to the difference in the explicit valence electron basis functions each basis set employs. At all other areas, the density agrees between the all-electron dev2-SVP calculation and the ECP Stuttgart calculation. In contrast, in the basis set where the Stuttgart ECP is used with the dev2-SVP basis, the double-peak feature disappears. The general shape of the density resembles the all-electron case while the overall density is reduced due to the ECP. As the dev2-SVP basis is not optimized with the Stuttgart ECP, it is not unexpected that electron density would be larger near the nucleus, but this may have consequences in the recovery of correlation energy by MC-PDFT.

**Figure 2:**
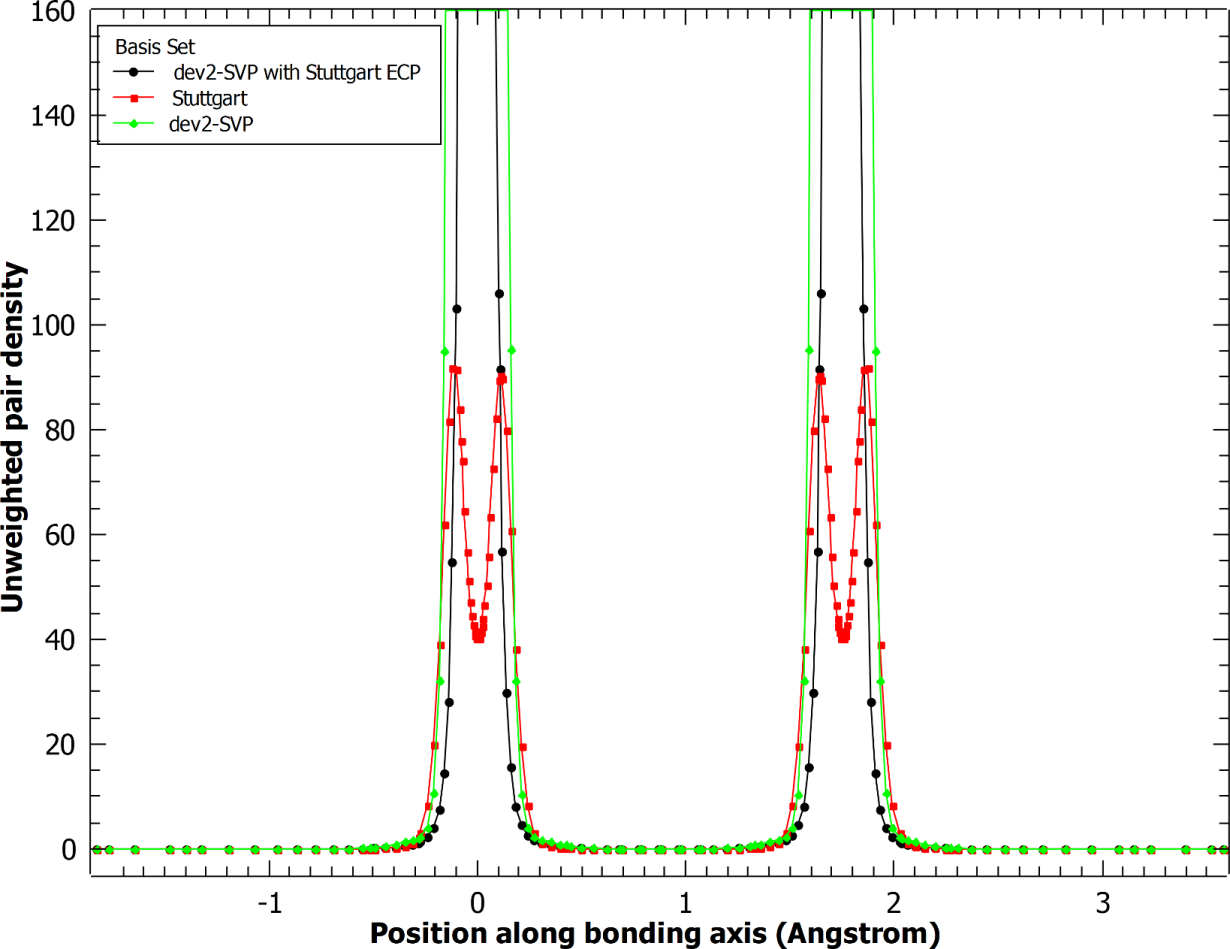
Unweighted electron pair density along the bonding axis for the chromium dimer with and without an effective core potential applied. Cr atoms are 1.75 Å apart.

Next, we examined the reference pair density (Figure 2). The pair density exhibits characteristics qualitatively similar to the one-electron density. The Stuttgart ECP basis set result shows a double-peak structure near each nucleus, while the all-electron and modified ECP basis set results show single peaks near each nucleus.

**Figure 3:**
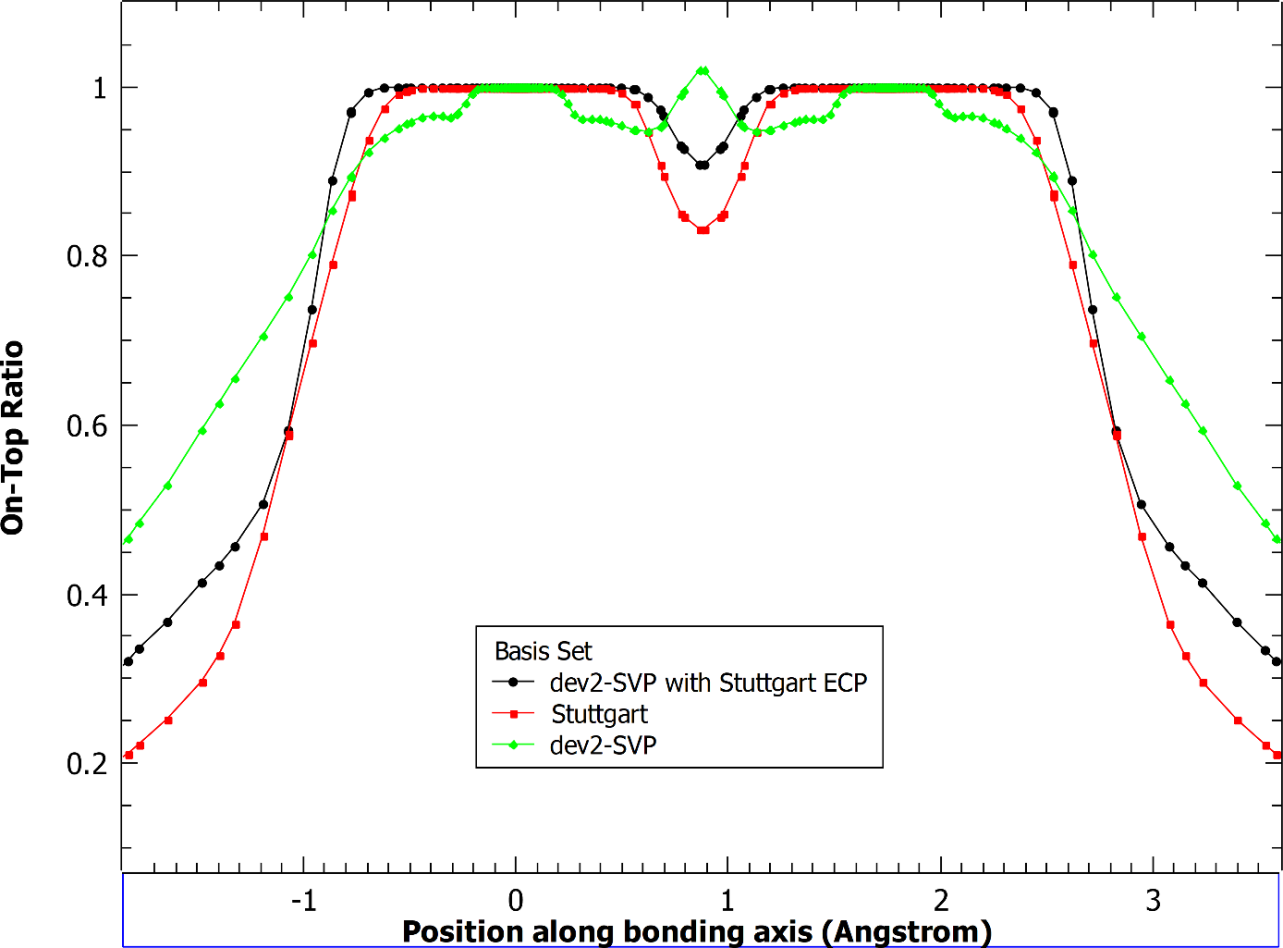
On-top ratio for the chromium dimer with and without an effective core potential applied. Cr atoms are 1.75 Å apart.

Using the density and pair density, we computed the on-top ratio (eq 13). This is the key quantity that determines the extent to which the on-top functional exhibits multiconfigurational (non-KS-DFT-like) character. Grid points with values different from 1 indicate where this occurs. The tPBE functional used in this work, which is formed by translating the α and β electron densities that are passed to the PBE functional, will not alter electron densities if the ratio is greater than or equal to 1, and pure-KS-DFT-like character is returned. The on-top pair density ratios for each of the explored basis sets are given in Figure 3.

We note that the all-electron basis (def2-SVP) has “flat” regions where the ratio is very close to one, centered around each atom. The width of this region is approximately 0.5 Å. The ECP basis sets employed are also flat in this range, suggesting that the on-top functional performs well very near the nucleus with the ECP. At a more intermediate distance (0.2–0.5 Å, for example), a sizable difference is seen between the Stuttgart ECP and all-electron result. This is the region where noticeable differences in electron density occurred. The ECP basis sets give larger flat regions, and dev2-SVP with Stuttgart ECP exhibits the largest flat region overall. This suggests that, because the dev2-SVP is not optimized to work with the Stuttgart ECP, this hybrid approach undercorrelates electrons due to significant electron density being expressed near the core, which the ECP should be preventing. With the unmodified Stuttgart ECP, agreement with the all-electron basis set is better. As discussed with the density, the deviations seen in the intermediate distances might be attributed to the use of the ECP, but they may be due to the difference in the explicit valence electron basis functions that each basis set employs.

**Figure 4:**
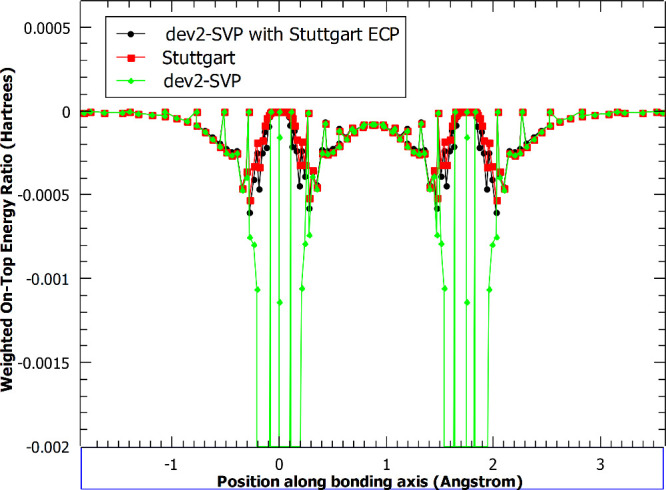
Weighted on-top energy for the chromium dimer with and without an effective core potential applied. Cr atoms are 1.75 Å apart.

In the region between the atoms, the all-electron basis set has a peak in the on-top ratio at the midpoint between the two atoms, whereas both ECP bases show a trough. Although there is a qualitative difference in the top-to-bottom ratios, this has a negligible effect on the energy contributions from these grid points. Figure 4 illustrates that the all-electron basis set and ECP basis sets all produce similar weighted energy contributions to the overall electronic energy. The largest and most important differences all appear very near to the nucleus, where agreement in the on-top ratios was best.

**Figure 5:**
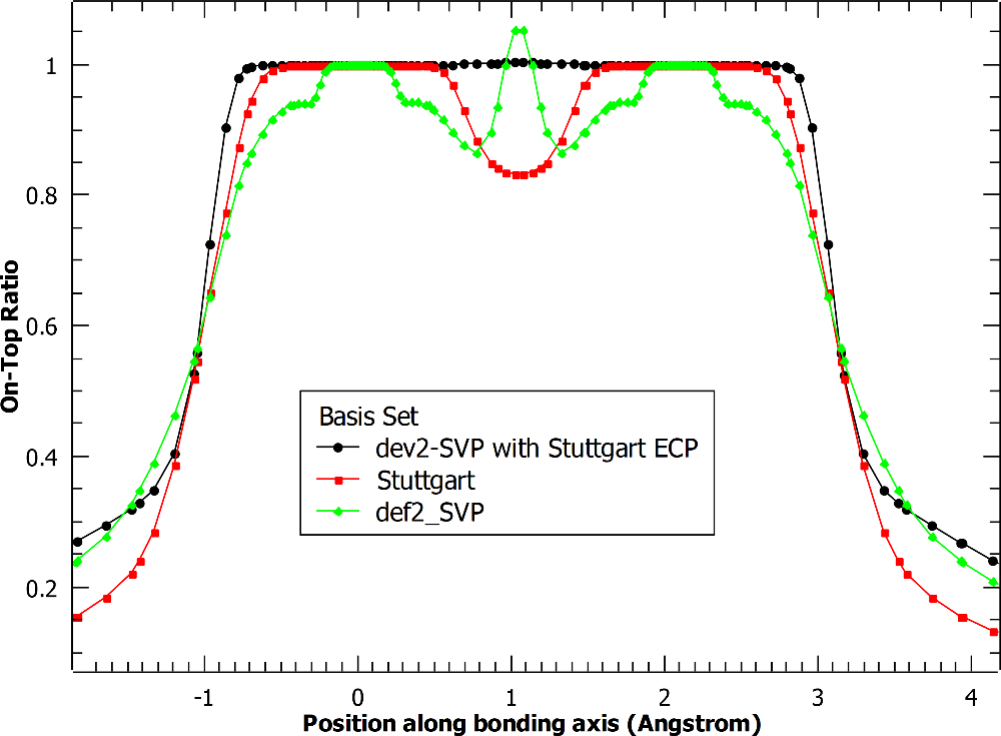
On-top ratio for the chromium dimer with and without an effective core potential applied. Cr atoms are 2.10 Å apart.

A similar analysis was performed on a stretched geometry. A bond distance of 2.10 Å was used. Plots of the electron and pair density can be found in the Supporting Information (Figures S1 and S2); they show qualitatively similar features to the previous 1.75 Å case. The top-on ratio is given in Figure 5. Like the equilibrium geometry, very similar features appear in the stretched case. The regions of space near the nuclei are noticeably flatter and wider for the ECP basis sets, and the midpoint between the nuclei has a difference in character for all-electron versus ECP basis sets. The similarity in performance between nonequilibrium stretched geometry and the equilibrium geometry results suggest that the description of bonding by PDFT may not be adversely affected when using an ECP basis set.

**Figure 6:**
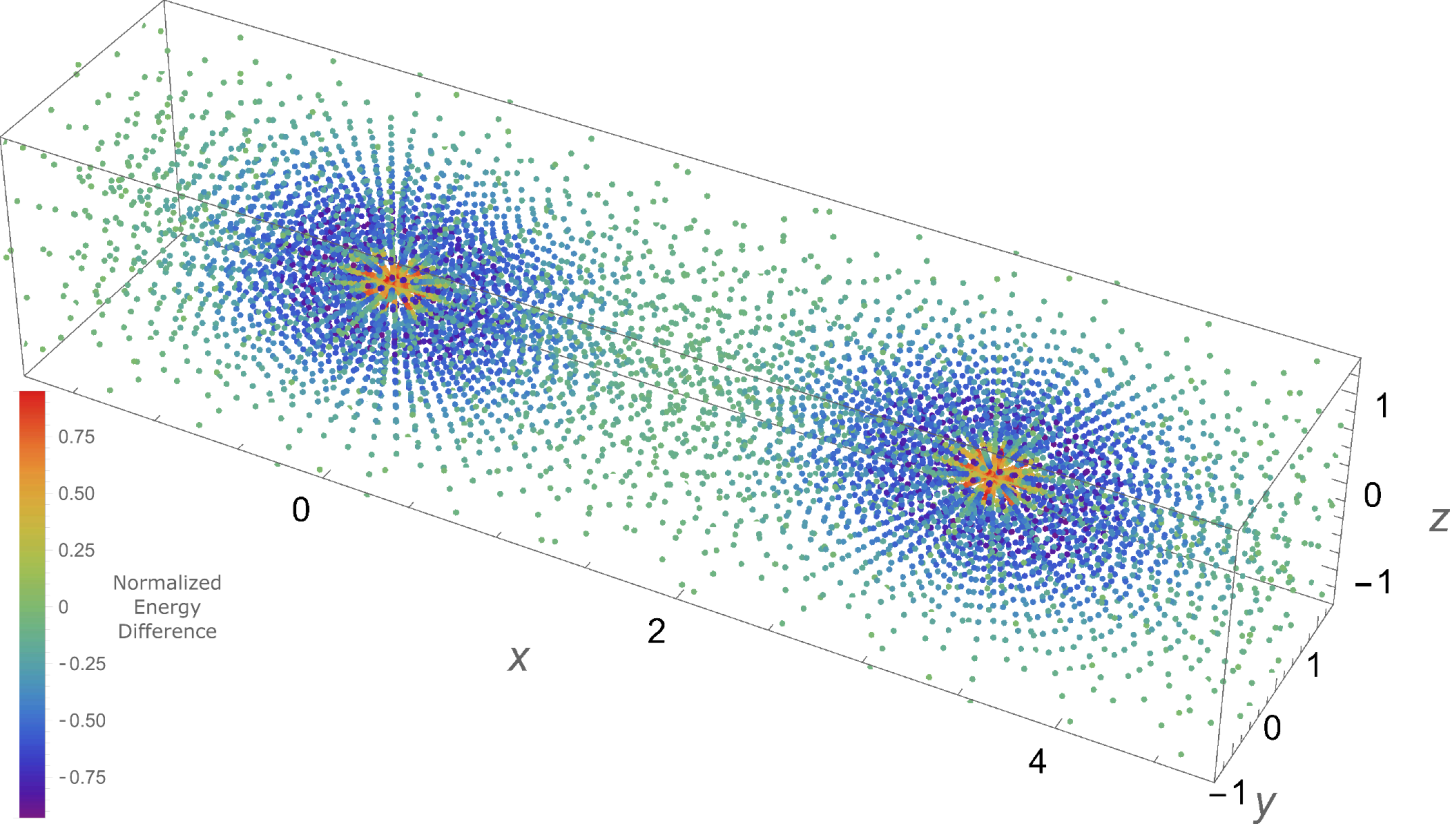
Relative contributions to the on-top energy from three-dimensional grid points for all-electron and ECP basis sets.

To further illustrate the differences in the handling of the on-top energy between the ECP and all-electron basis sets, a plot of the on-top energy in the three-dimensional grid space around the chromium dimer system was generated. A nuclear separation distance of 3.0 Å was used, and the all-electron def2-TZVP basis and the ECP Stuttgart basis set were used. Figure 6 illustrates the relative importance of each grid point for ECP vs all-electron bases. To generate this plot, the values of the energy contribution from each grid point to the on-top energy were computed, and each set was normalized using a minimum-maximum feature scaling scheme. The normalized energy contribution of the ECP basis set was then subtracted from the normalized energy contribution of the all-electron basis set at each point, giving an associated value of the normalized on-top energy contribution difference between the all-electron and ECP basis sets. Points with values between zero and 1 indicate locations where the all-electron basis set make larger relative contributions to the overall on-top energy, and points with values between −1 and 0 indicate locations where the ECP basis set make larger relative contributions to the overall on-top energy. Examination of the plot shows that, in regions of space far from the nucleus, contributions at the grid points to the overall energy are similar between the ECP and all-electron basis set, and these contributions are very small. Close to the nuclei, however, the effect of the ECP is strongly seen for the all-electron basis set; these points give a very large contribution to the overall on-top energy. In contrast, the ECP basis set yields a very small to negligible energy contribution. In the intermediate regions of space, near the atoms but outside of the effective core, the plot illustrates that these points contribute most strongly to the total on-top energy with the ECP basis set. To summarize the results of Figures 3 and 6, the on-top energy contribution determined in an MC-PDFT calculation with an ECP basis set has a much smaller overall magnitude than an all-electron basis calculation, and it is the points just outside of the atomic core regions that contribute most strongly.

### 4.2 Test system: The Copper Dimer

The results of the previous section illustrated that using an ECP with MC-PDFT limits the regions in space where the on-top density functional can have an effect on the overall energy description. This is not unexpected; an ECP is designed to simplify the description of the electronic structure of a chemical system. However, commonly used ECP basis sets are parametrized from calculations different than MC-PDFT. Thus, we seek to explore how these common ECP basis sets perform for chemical systems relative to all-electron basis sets with the use of MC-PDFT.

**Figure 7:**
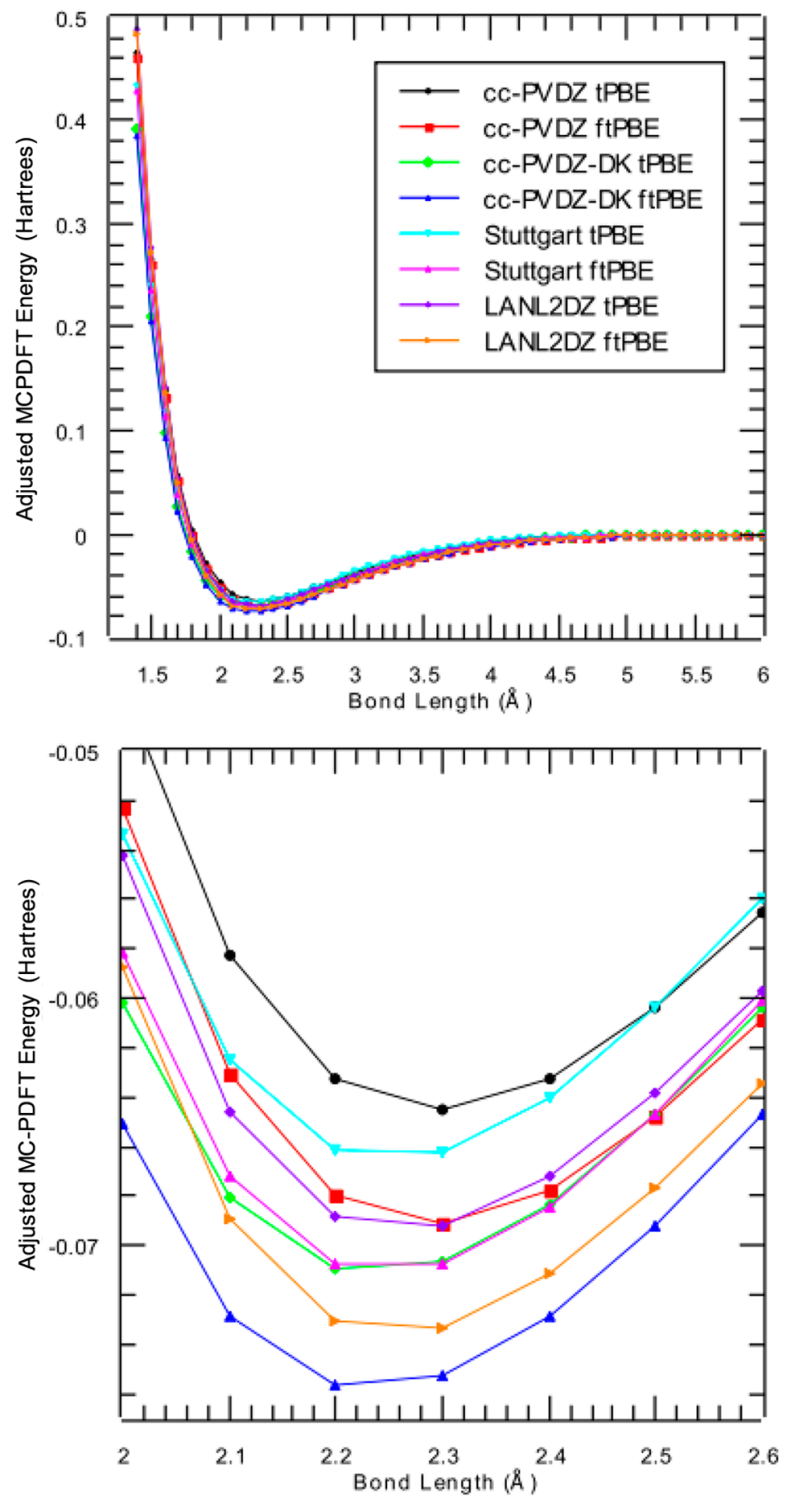
Dissociation of the copper dimer for several all-electron and ECP basis sets.

To explore a full bond dissociation curve, the Cu dimer was used as a test case. A series of calculations were performed, all employing a (2e, 2o) basis set. Two functionals were explored (tPBE, ftPBE). Nonrelativistic all-electron calculations were done with the cc-pVDZ basis set, and relativistic all-electron calculations with the Douglas-Kroll-Hess Hamiltonian were done using the cc-pVDZ-DK basis set. The Stuttgart (relativistic) and LANL2DZ (nonrelativistic) ECP basis sets were also used. The bond dissociation curves are presented in Figure 7. Qualitatively, all curves agree with an equilibrium bond distance between 2.2 and 2.3 Å. The all-electron cc-pVDZ-DK relativistic calculation did the best at reproducing the experimental dissociation energy (0.081 ± 0.004 hartree^46^), and the best ECP basis set results were seen when the ftPBE functional was used. While the LANL2DZ result obtains a closer dissociation energy, the Stuttgart basis curve better represents the shape of the all-electron result. Comparing the nonrelativistic calculations (cc-pVDZ and LANL2DZ), the ftPBE functional predicts a deeper well depth than the tPBE functional for both classes of basis sets. The same trend is noted in the relativistic calculations (cc-pVDZ-DK and Stuttgart).

### 4.3 Ionization Potentials of First-Row Transition Metal-Containing Diatomic Systems

The accurate determination of physical molecular properties is important for computational methods. To test the ability of MC-PDFT to reproduce experimental results when using ECP basis sets, we calculated ionization potentials for a wide set of first-row transition metal-containing diatomic systems. We employed a mixed-basis set approach; we used an ECP basis set for transition metal atoms (Stuttgart or LANL2DZ) and used the maug-cc-pVDZ basis set for nontransition metal atoms. The tPBE PDFT functional was used for all calculations. We compared our results to both experimental results and computational KS-DFT results from the literature. Overall, 26 systems were selected (Table 1) from a larger set of molecules previously explored using a variety of DFT functionals and a mixed all-electron/ECP basis set approach.^33^ We limited our test set to diatomic systems in order to minimize any role that molecular geometries might have on the results. These selected systems included a variety of early- and late-first-row transition metals, and the systems can be subdivided into hydrides, chalogenides/oxides, metal dimers, and halides. Active spaces [(number of electrons, number of orbitals)] were chosen to include all valence electrons and valence atomic orbital character from the transition metal in addition to important bonding and antibonding orbital character from the nonmetal ligands. For each system, the geometry was optimized for both the neutral and cation species for multiple spin states, and the energy difference between the lowest-energy spin states of the neutral and cation species were used to determine the ionization energy. The two basis set selections agreed on the lowest spin state for all calculations except for CoH^+^, where the Stuttgart basis set predicted a spin mulitiplicity of 4 whereas the LANL2DZ basis set predicted a spin multiplicity of 2. The calculated energy differences for LANL2DZ between these two spin states were small (0.008 hartree). Equilibrium geometries and absolute energies are available in Tables S1–S2.

**Table 1:** Active Space Parameters (Electron, Orbitals) for Each Calculation^a^

		Spin Multiplicity	
Molecule	Active Space (e, o)	Neutral	Cation
TiH	(5, 7)	4	3
TiO	(8, 9)	3	2
TiS	(8, 9)	3	2
V_2_	(10, 12)	3	4
VO	(9, 9)	4	3
VS	(9, 9)	4	3
VN	(8, 9)	3	2
CrO	(10, 9)	5	4
CrF	(7, 7)	6	5
CrCl	(7, 7)	6	5
MnF	(10, 8)	7	6
MnCl	(10, 8)	7	6
MnH	(8, 8)	7	6
MnO	(11, 9)	6	7
Fe_2_	(16, 12)	9	8
FeCl	(9, 7)	6	5
FeO	(12, 9)	5	6
CoH	(10, 7)	3	4^b^ or 2^c^
CoO	(13, 9)	4	5
CoCl	(10, 7)	3	4
NiCl	(11, 7)	2	3
NiH	(11, 7)	2	3
NiO	(14, 9)	3	4
Cu_2_	(22, 12)	1	2
CuCl	(12, 7)	1	2
CuF	(12, 7)	1	2

a The lowest-energy spin multiplicities for each system are listed.

b cc-pVDZ + Stuttgart.

c cc-pVDZ + LANL2DZ.

**Figure 8:**
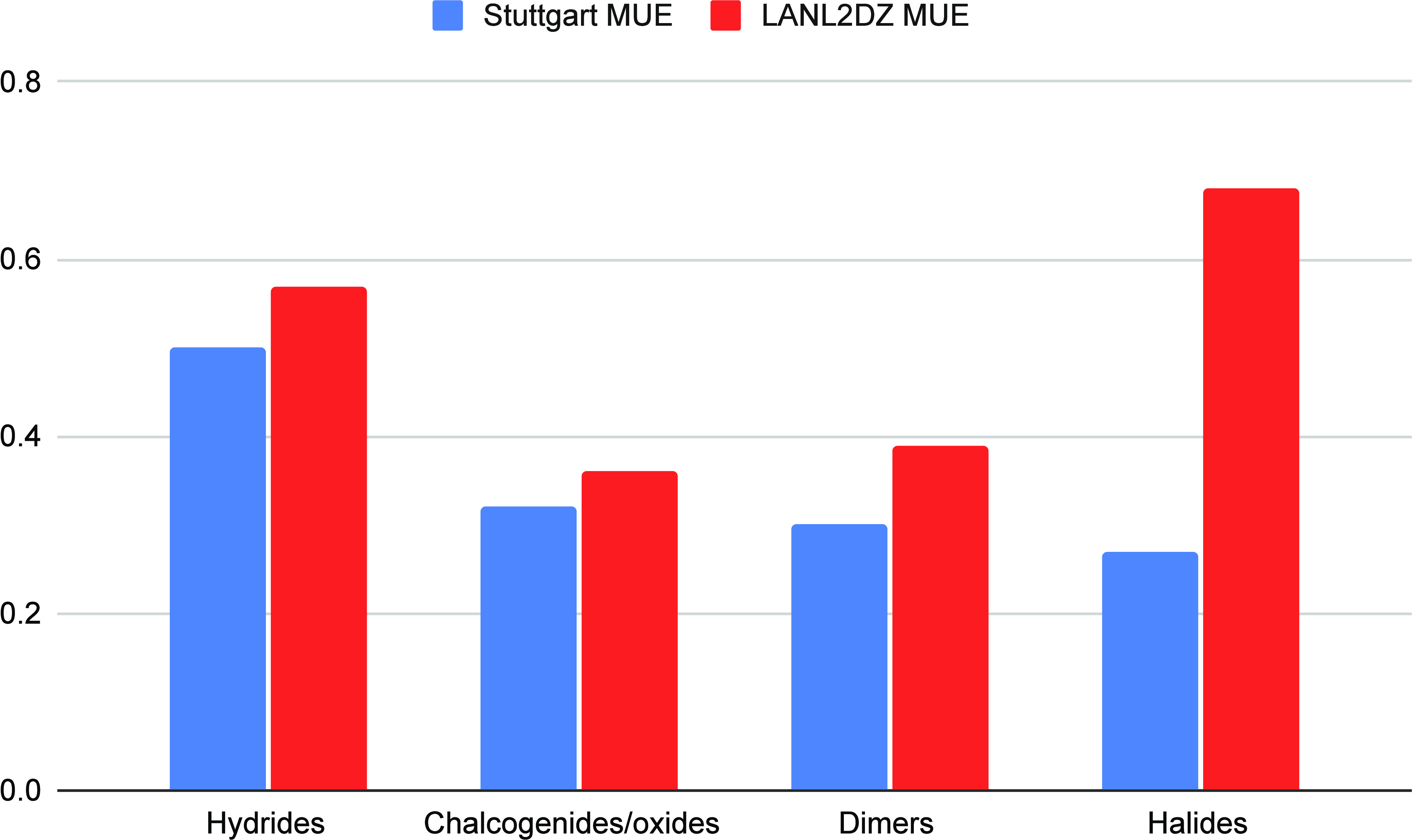
Mean unsigned errors of ionization energies by category. Units are given in eV.

Computed results for the ionization energies are given in Table 2, and Figure 8 shows the mean unsigned error for the dimer calculations separated by molecule type. In all categories, the Stuttgart basis set did a better job at reproducing the experimental ionization energies than did the LANL2DZ basis set. For the hydrides, chalcogenides, oxides, and dimers, the Stuttgart basis typically provided ionization energies that were 0.1 eV more accurate, on average. The halides showed the greatest disparity. The Stuttgart basis set resulted in errors that were very similar to those in the other categories, but the LANL2DZ basis set errors were much larger.

**Table 2:** Ionization Energies of Selected First-Row Transition Metal Diatomic Systems^a^

System	Experimental	I.E. Stutt.	I.E. LANL2DZ	Error Stutt.	Error LANL2DZ
TiH	6.0	6.69	6.21	0.69	0.21
TiO	6.82	7.06	6.99	0.24	0.17
TiS	7.1	7.20	7.16	0.10	0.06
V_2_	6.36	6.46	7.16	0.10	0.80
VO	7.46	7.52	7.42	0.06	–0.04
VS	7.69	8.00	8.06	0.31	0.37
VN	7.27	7.4	7.21	0.13	–0.06
CrO	8.16	7.96	8.07	–0.20	–0.09
CrF	9.3	8.41	8.09	–0.89	–1.21
CrCl	8.5	8.50	9.11	0.00	0.61
MnF	8.51	8.53	7.99	0.02	–0.52
MnCl	8.5	7.86	7.81	–0.64	–0.69
MnH	7.8	6.85	6.81	–0.95	–0.99
MnO	8.65	9.23	8.13	0.58	–0.52
Fe_2_	6.3	6.76	6.59	0.46	0.29
FeCl	8.08	8.09	7.94	0.01	–0.14
FeO	8.9	8.63	8.29	–0.27	–0.61
CoH	7.86	7.91	8.05	0.05	0.19
CoO	8.9	8.63	8.32	–0.27	–0.58
CoCl	8.71	8.31	8.04	–0.40	–0.67
NiCl	9.28	9.05	10.23	–0.23	0.95
NiH	8.5	8.88	9.13	0.38	0.63
NiO	9.5	8.97	8.86	–0.53	–0.64
Cu_2_	7.9	8.23	7.98	0.33	0.08
CuCl	10.7	10.60	10.44	–0.10	–0.26
CuF	10.9	10.75	11.47	–0.15	0.57
MSE				0.04	0.08
MUE				0.31	0.46

a Energies are given in eV.

Some of the greatest errors for the LANL2DZ basis set are in systems that contain either fluorine or chlorine. Some transition metal halides, notably CuCl, are important materials for optoelectronic devices,^32^ and accurate predictions of their fundamental properties are important. Struggles with ionization energy predictions for fluorine-containing species have been noted before in a previous work using a mixed 6-31+G** + LANL2DZ basis set approach with various DFT functionals,^33^ so this result may be endemic to the use of the LANL2DZ basis set for this class of systems. Interestingly, the Stuttgart basis set performed very well for this class of system, having the lowest mean unsigned error in this category compared with all other categories.

Because an MC-PDFT calculation generally requires a multiconfigurational reference wave function calculation (which can be expensive), one would hope to see an improvement in predicted ionization energies compared to those obtained via KS-DFT calculations. Table 3 compares the Stuttgart and LANL2DZ results against DFT results on the same species from a previous study.^33^ Overall, Stuttgart performed the best, with lower MSE and MUE compared to LANL2DZ or the DFT results from the literature. The MC-PDFT LANL2DZ calculations exhibited errors similar to those found in the previous KS-DFT study.

**Table 3:** Mean Signed Errors and Mean Unsigned Errors for All Systems Explored^a^

Method	MSE	MUE
btPBE (cc-PVDZ + Stuttgart)	0.04	0.31
btPBE (cc-pVDZ + LANL2DZ)	0.08	0.46
cYang VIE BLYP	0.10	0.36
cMPWPW91	0.04	0.34
cPBEPBE	0.08	0.32
cB3LYP	0.09	0.44
cPBE1PBE	0.17	0.51
cB98	0.21	0.50
cTPSSTPSS	0.22	0.39
cTPSSKCIS	0.20	0.37
cBB95	–0.08	0.70
cB1B95	0.15	0.62
cTPSS1KCIS	0.29	0.45
cBB1K	–0.18	0.93

a Energy errors are given in eV.

b This work.

c From ref (33) using 6-31+G** + LANL2DZ.

## 5 Conclusions

In this work, we have illustrated the effect that an ECP basis set has in the determination of the on-top energy and have explored its effect on the determination of several molecular properties. The use of an ECP, by removing explicit electronic density, does affect the ability of the most widely used MC-PDFT on-top functionals to recover and describe electron correlation. This is not unexpected or surprising (ECPs are approximations developed to describe some degree of electron interactions), but we explicitly showed the effects that the use of an ECP has on the determination of the on-top energy of the remaining valence electrons as part of an MC-PDFT calculation. By removing explicit electron density with an ECP, larger regions of three-dimensional space no longer have on-top ratios that differ significantly from 1, and the translated class of on-top functionals will behave in a manner more similar to the traditional KS-DFT functional from which they are derived, perhaps at distances further from the atomic cores than would have been expected. As many previous studies have noted, it may be fortuitous or necessary to devise method-specific ECPs to obtain a level of chemical accuracy desired for a particular study.^26−28^

Despite these concerns, ECP and mixed-ECP approaches are often employed with methods outside of which the original ECP was parametrized with. We explored this approach using MC-PDFT. We found overall good descriptions of the geometry and energetics of the bond dissociations. For ionization energies of transition-metal containing dimers, we found that a cc-pVDZ+Stuttgart approach returned energies more in line with the experimental results, with mean-signed mean-unsigned errors of 0.04 and 0.31 eV, respectively, across the testing set. The maug-cc-pVDZ+LANL2DZ approach performed slightly worse, with mean-signed mean-unsigned errors of 0.11 and 0.46 eV, respectively, across the testing set, but similar to errors found from KS-DFT approaches.

As MC-PDFT is applied to larger and more complicated systems (molecular electronics, for example^37^), the use of ECPs will be essential in keeping computational costs manageable. Off-the-shelf ECP approaches may be sufficient for these studies, but two possible approaches may also be developed: parametrization of new ECP basis sets specifically for existing MC-PDFT functionals or the development of new functionals that explicitly consider core electrons in a more nuanced way.

## Supporting Information Available

Unweighted electron and electron pair density along the bonding axis for the chromium dimer; computed equilibrium geometries and absolute energies for transition-metal-containing dimers computed with MC-PDFT (PDF)
