# Supplementary material for: The Use of Effective Core Potentials with Multiconfiguration Pair-Density Functional Theory
Source: J Phys Chem A. 2024 Jul 25;128(31):6555–65. doi: 10.1021/acs.jpca.4c02666 (PMC11317981; doi:10.1021/acs.jpca.4c02666)
Supplement: Supplementary file 1 — jp4c02666_si_001.pdf [file jp4c02666_si_001.pdf]

SUPPORTING INFORMATION

The Use of Effective Core Potentials with Multiconfiguration Pair-Density Functional Theory

William E. Minnette, III,[†] Erik P. Hoy,[‡] and Andrew M. Sand[†]

† *Department of Chemistry and Biochemistry, Butler University, Indianapolis, Indiana 46208, United States*

‡ *Department of Chemistry – Rowan University, Glassboro, New Jersey 08028, United States*

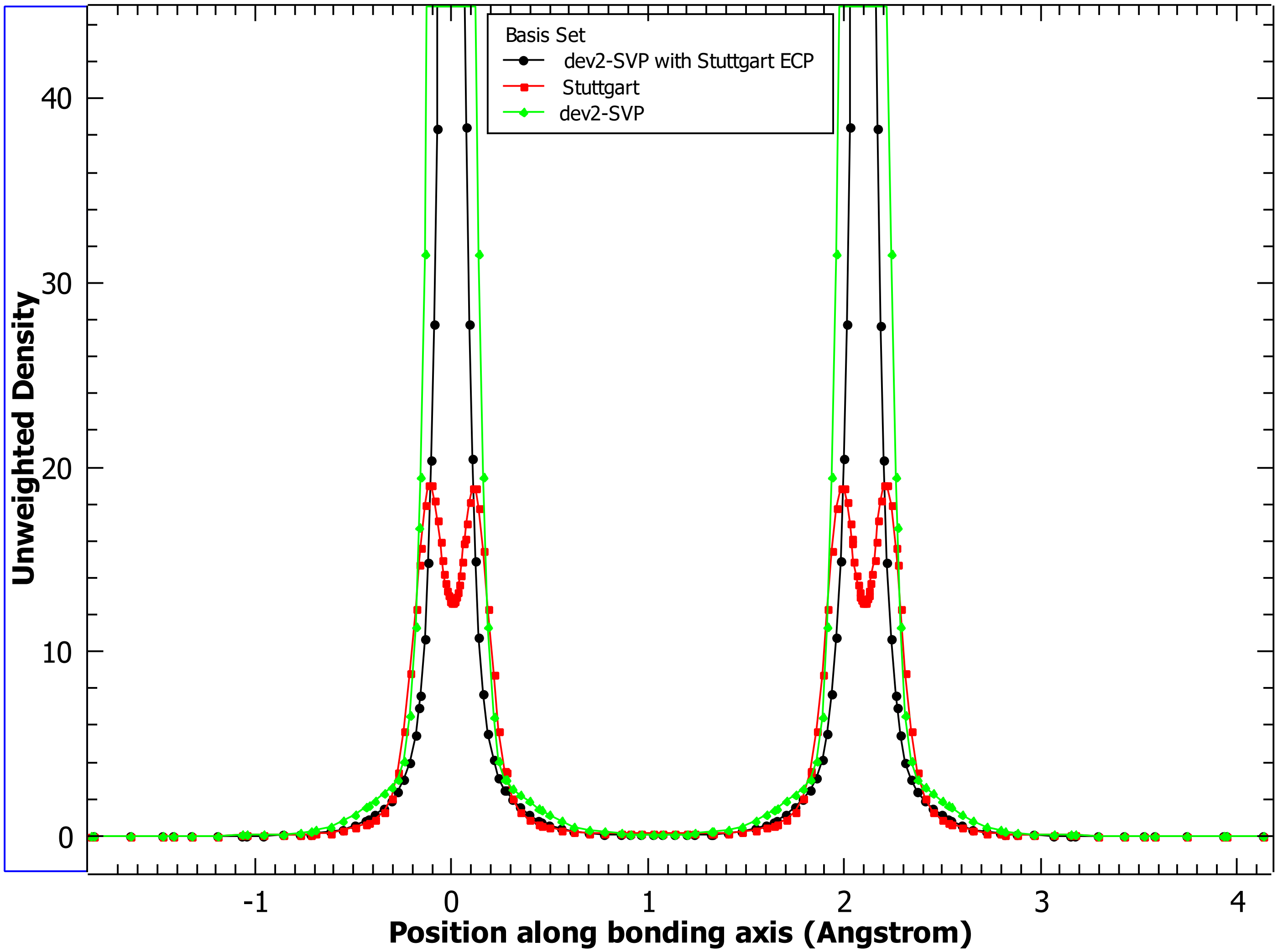

Figure S1: Unweighted electron density along the bonding axis for the chromium dimer, with and without an effective core potential applied. Cr atoms are 2.10 Å apart.

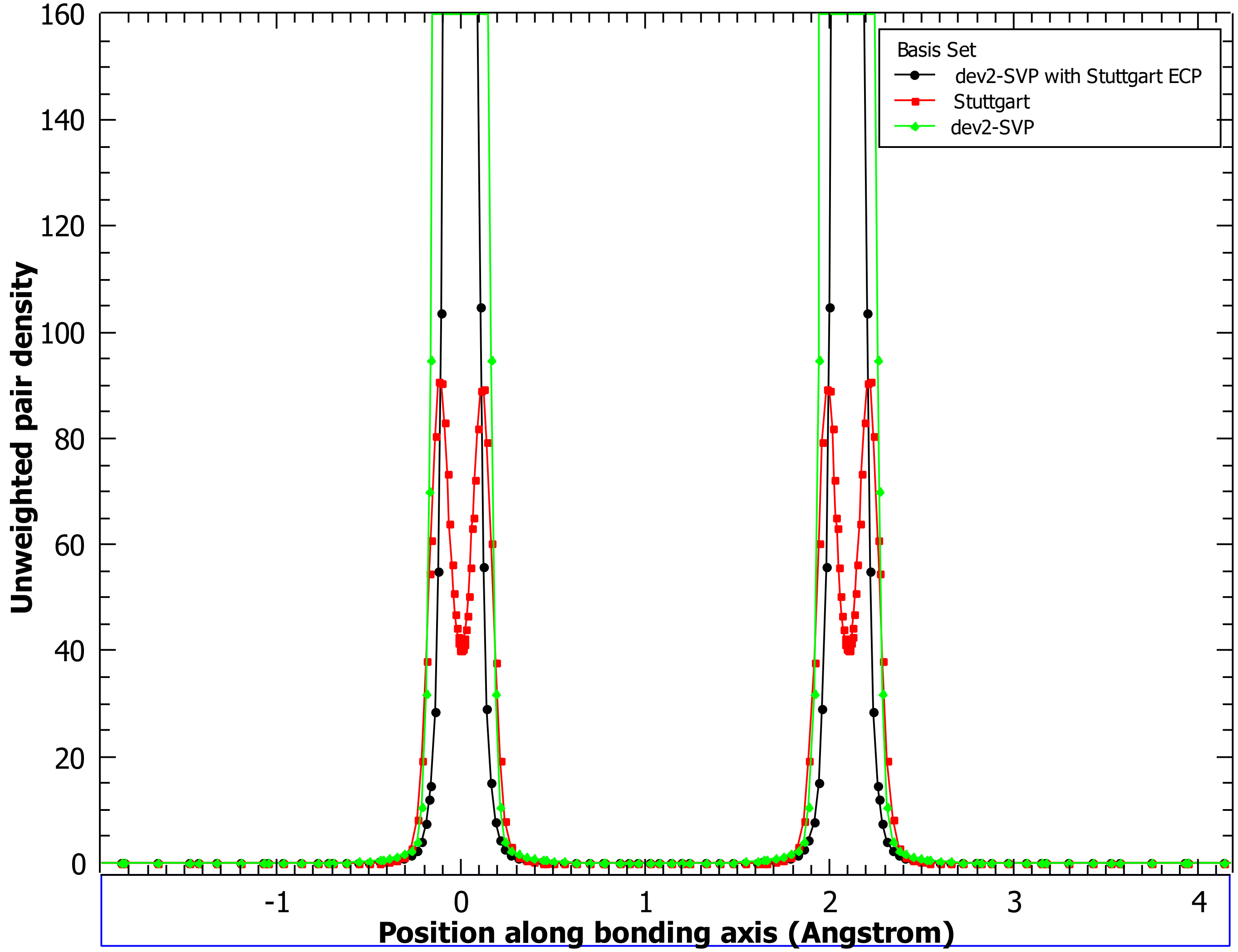

Figure S2: Unweighted electron pair density along the bonding axis for the chromium dimer, with and without an effective core potential applied. Fr atoms are 2.10 Å apart.

Table S1: Computed equilibrium geometries and absolute energies for transition-metal-containing dimers computed with MC-PDFT (tPBE/maug-cc-pVDZ + Stuttgart).

	Neutral				Cation		
System	Bond distance (Å)	Active space (e,o)	Spin multiplicity	Energy (hartrees)	Active space (e,o)	Spin multiplicity	Energy (hartrees)
TiH	1.714	(5,7)	4	-58.744650	(4,7)	3	-58.498956
TiO	1.618	(8,9)	3	-133.412450	(7,9)	2	-133.153012
TiS	2.096	(8,9)	3	-456.279796	(7,9)	2	-456.015254
V_2	1.951	(10,12)	3	-143.146404	(9,12)	4	-142.909084
VO	1.593	(9,9)	4	-146.768778	(8,9)	3	-146.492457
VS	2.071	(9,9)	4	-469.641440	(8,9)	3	-469.347444
VN	1.574	(8,9)	3	-126.243856	(7,9)	2	-125.971836
CrO	1.624	(10,9)	5	-162.055124	(9,9)	4	-161.762570
CrF	1.762	(7,7)	6	-186.701016	(6,7)	5	-186.391985
CrCl	2.139	(7,7)	6	-546.968627	(6,7)	5	-546.656301
MnF	1.866	(10,8)	7	-204.075997	(9,8)	6	-203.762432
MnCl	2.287	(10,8)	7	-564.350350	(9,8)	6	-564.061428
MnH	1.743	(8,8)	7	-104.840783	(7,8)	6	-104.589140
MnO	1.610	(11,9)	6	-179.401701	(10,9)	7	-179.062644
Fe_2	2.417	(16,12)	9	-247.634955	(15,12)	8	-247.386661
FeCl	2.226	(9,7)	6	-583.870266	(8,7)	5	-583.573124
FeO	1.576	(12,9)	5	-198.933659	(11,9)	6	-198.616628
CoH	1.511	(10,7)	3	-146.294405	(9,7)	4	-146.003776
CoO	1.616	(13,9)	4	-220.840592	(12,9)	5	-220.523303

CoCl	2.109	(10,7)	3	-605.754806	(9,7)	4	-605.449524
NiCl	1.818	(11,7)	2	-630.874561	(10,7)	3	-630.542019
NiH	1.446	(11,7)	2	-171.424893	(10,7)	3	-171.098466
NiO	1.564	(14,9)	3	-245.933360	(13,9)	4	-245.603676
Cu_2	2.251	(22,12)	1	-394.634305	(21,12)	2	-394.331732
CuCl	2.068	(12,7)	1	-657.356771	(11,7)	2	-656.967116
CuF	1.766	(12,7)	1	-297.017879	(11,7)	2	-296.622880

Table S2: Computed equilibrium geometries and absolute energies for transition-metal-containing dimers computed with MC-PDFT (tPBE/maug-cc-pVDZ + LANL2DZ).

	Neutral				Cation		
System	Bond distance (Å)	Active space (e,o)	Spin multiplicity	Energy (hartrees)	Active space (e,o)	Spin multiplicity	Energy (hartrees)
TiH	1.683	(5,7)	4	-58.505114	(4,7)	3	-58.276809
TiO	1.630	(8,9)	3	-133.166585	(7,9)	2	-132.909698
TiS	2.090	(8,9)	3	-456.038958	(7,9)	2	-455.775919
V2	3.369	(10,12)	3	-142.468828	(9,12)	4	-142.205525
VO	1.607	(9,9)	4	-146.408132	(8,9)	3	-146.135518
VS	2.076	(9,9)	4	-469.286498	(8,9)	3	-468.990402
VN	1.577	(8,9)	3	-125.885045	(7,9)	2	-125.619999
CrO	1.658	(10,9)	5	-161.360558	(9,9)	4	-161.063990
CrF	1.777	(7,7)	6	-186.007314	(6,7)	5	-185.710141

CrCl	2.226	(7,7)	6	-546.314454	(6,7)	5	-545.979571
MnF	1.864	(10,8)	7	-203.624693	(9,8)	6	-203.330900
MnCL	2.280	(10,8)	7	-563.897926	(9,8)	6	-563.611076
MnH	1.717	(8,8)	7	-104.392048	(7,8)	6	-104.141657
MnO	1.821	(11,9)	6	-178.915390	(10,9)	7	-178.616768
Fe2	2.460	(16,12)	9	-246.657287	(15,12)	8	-246.415105
FeCl	2.231	(9,7)	6	-583.365527	(8,7)	5	-583.073889
FeO	1.583	(12,9)	5	-198.435971	(11,9)	6	-198.131220
CoH	1.331	(10,7)	3	-145.528111	(9,7)	2	-145.232257
CoO	1.658	(13,9)	4	-220.054165	(12,9)	5	-219.748580
CoCl	2.091	(10,7)	3	-604.975353	(9,7)	4	-604.679916
NiCl	1.818	(11,7)	2	-629.264460	(10,7)	3	-628.888665
NiH	1.457	(11,7)	2	-169.7880681	(10,7)	3	-169.4526925
NiO	1.583	(14,9)	3	-244.287530	(13,9)	4	-243.962031
Cu2	2.263	(22,12)	1	-392.247839	(21,12)	2	-391.954548
CuCl	2.101	(12,7)	1	-656.161079	(11,7)	2	-655.777589
CuF	1.800	(12,7)	1	-295.858951	(11,7)	2	-295.437423
